# Same Day Discharge After Prostatectomy for Prostate Cancer and Readmissions

**DOI:** 10.1002/cam4.71564

**Published:** 2026-02-13

**Authors:** Christopher P. Dall, Xiu Liu, Sarah J. Leick, Preeti Chachlani, Kassem S. Faraj, Arnav Srivastava, Samuel R. Kaufman, Vahakn B. Shahinian, Brent K. Hollenbeck

**Affiliations:** ^1^ Department of Urology Massachusetts General Hospital Boston Massachusetts USA; ^2^ Department of Urology Brigham and Women's Hospital Boston Massachusetts USA; ^3^ Division of Health Services Research, Department of Urology University of Michigan Ann Arbor Michigan USA; ^4^ Division of Nephrology, Department of Internal Medicine University of Michigan Ann Arbor Michigan USA

**Keywords:** discharge, outcomes, prostate, readmission

## Abstract

**Background:**

Same‐day discharge following radical prostatectomy has become increasingly common, with single‐institution series suggesting it reduces healthcare costs without increasing adverse events. However, this practice has not been studied nationally, outside of specialized centers. This study assesses 30‐day readmissions, observation stays, and emergency department visits among men with prostate cancer undergoing prostatectomy.

**Study Design:**

We used national Medicare data to identify men undergoing prostatectomy for prostate cancer between 2016 and 2021. We focused on patients discharged either the same day or the day after surgery to include only those with an uneventful postoperative course presumably eligible for same‐day discharge. We used multivariable logistic regression to measure relationships between discharge day (same‐day vs. next‐day) and 30‐day readmissions, adjusted for patient factors. We also assessed the association between the day of discharge and a secondary outcome, a composite of readmission, observation stay, or emergency department visits within 30 days.

**Results:**

Our cohort included 528 men discharged the same day and 11,513 discharged the next day. By 2021, same‐day discharges rose to 9.2%. Same‐day discharge was associated with an almost two‐fold increase in the odds of a readmission within 30 days (adjusted OR: 1.93; 95% CI 1.35–2.76; *p* < 0.01). However, the odds of an acute care event, measured by a composite of any readmission, observation stay, or emergency department visit, were similar in both groups (adjusted OR: 1.16; 95% CI 0.90–1.50; *p* = 0.27).

**Conclusions:**

Same‐day discharges after prostatectomy have increased substantially but were associated with a two‐fold increase in odds of a readmission within 30 days. However, global adverse events, as measured by our composite outcome, were similar.

## 1 Introduction

Prostate cancer surgery is common, with more than 60,000 procedures performed annually [1]. The dissemination of minimally invasive approaches, and robotic surgery in particular, has been associated with shorter lengths of stays and decreased blood loss [2, 3, 4]. With the removal of radical prostatectomy from the Centers for Medicare and Medicaid Services' “inpatient only” list in 2018 [5] and pressure to avoid hospitalization during the COVID‐19 pandemic, some surgeons are discharging their patients the day of surgery [6, 7, 8, 9]. Though limited to small series, prior work suggests that same‐day discharge is safe and feasible in select patients, with similar complication and readmission rates [6].

The extent to which same‐day discharge after prostatectomy for prostate cancer has gained traction nationally is unknown. Further, the generalizability of the safety of this approach outside selected institutions with deep experience in managing these patients is also uncertain. On one hand, surgeon skill and knowledge in selecting appropriate candidates for same day discharge may be uniform and readily transferable, supporting expansion of this approach. This would reduce unnecessary hospitalization and associated resource utilization while simultaneously freeing hospital beds for those with greater need. On the other hand, prostate cancer commonly occurs in the Medicare population [10], a cohort with typically more underlying health issues and medication requirements [11, 12], which may increase alterations in normal physiology in the postoperative setting. Finally, the severity of coexisting illnesses are heterogeneous. Because the severity of underlying conditions may not be fully appreciated by the surgeon, suitability for same day discharge may be difficult, or even impossible, to predict with precision. Insufficient post‐surgical monitoring and/or education, unpredictable responses to anesthesia affecting patient behavior, and reduced social support [13] have the potential to amplify the risk of adverse events in this population.

To better understand this issue, we performed a study measuring readmissions after prostatectomy for prostate cancer using national Medicare data. At this early phase of dissemination of same‐day discharge, we would expect surgeons to be conservative in who they deemed appropriate candidates. Thus, we hypothesized that readmissions would be similar between men discharged on the day of surgery and those discharged on the day after surgery.

## 2 Methods

We used a 20% national sample of fee‐for‐service Medicare patients, aged 66 and older, undergoing prostatectomy for prostate cancer between 2016 and 2021 from both inpatient and outpatient settings. Men were identified using International Classification of Diseases, Tenth revision, diagnosis and procedure codes as well as Healthcare Common Procedure Coding system codes. Patients who were age 65 (*n* = 2174) or who did not have Part A and B enrollment for 12 months prior to surgery (*n* = 377) were excluded to assess comorbidity for the 12‐month period prior to surgery. We further limited the study to men discharged on the day of surgery (i.e., same‐day) or the day after surgery (i.e., next‐day) (*n* = 15,330), with the day of discharge serving as the exposure. This excluded 6198 men discharged on or after postoperative day 2 to exclude those with perioperative complications or those requiring longer hospitalizations for medical and/or social reasons. Two patients died in the initial hospitalization and 50 patients with missing records were excluded from analysis. All men were followed for at least 30 days postoperatively and thus discharges occurring in the last quarter of 2021 were excluded (*n* = 686).

### 2.1 Outcomes

The primary outcome was a readmission to any hospital within 30 days of discharge following a prostatectomy for prostate cancer. We hypothesized that there would be no difference in readmissions between same‐day and next‐day discharges. As a secondary outcome, we defined a composite measure of requiring acute care inclusive of a visit to the emergency department, a hospital observation stay, or a readmission. These events were mutually exclusive, such that if a patient experienced a readmission, they were not categorized as having an observation stay or ED visit. The hierarchy of assessing the composite measure, in order of precedence, was readmission, observation stay, and emergency department visit.

### 2.2 Statistical Analysis

Differences between patients based on day of discharge were assessed using *t*‐tests and chi‐square tests for continuous and categorical data, respectively. Multivariable logistic regression was used to determine the relationship between prostatectomy day of discharge (same day vs. next day) and readmission within 30 days of discharge. The patient was the unit of analysis. The model was adjusted for patient age, dual eligibility, surgical approach (minimally invasive vs. open), race, year of surgery, and comorbidity, which was defined using the Charlson Comorbidity Index [14]. A similar modeling framework was used to measure the association between prostatectomy day of discharge and the composite measure of requiring acute care within 30 days of discharge.

SAS statistical software (version 9.4, SAS Institute Inc.) was used for all analyses. All tests were two‐sided, and the probability of type 1 error (α) was set at 0.05. The study protocol was deemed exempt from review by the institutional review board.

## 3 Results

**FIGURE 1:**
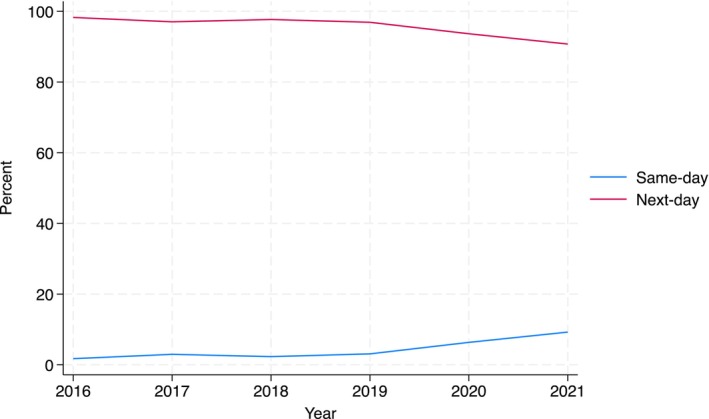
Percentage of same‐day and next‐day prostatectomy discharges.

Table 1 illustrates patient characteristics, stratified by day of discharge. There were no significant differences in age, race, comorbidity, or dual eligibility between patients discharged the same‐day versus next‐day. As illustrated in Figure 1, the percentage of patients discharged the same day increased annually (*p* < 0.01). In 2016, only 1.7% of patients were discharged the same day compared to 9.2% in 2021.

**TABLE 1:** Patient characteristics.

	Same‐day discharges (*n* = 528)	Next‐day discharges (*n* = 11,513)	*p*
Age, mean (SD)	70.62 (3.33)	70.54 (3.24)	0.56
Race			
White	442 (83.71%)	9779 (84.94%)	0.25
Black	37 (7.01%)	683 (5.93%)	
Other	11 (2.08%)	361 (3.14%)	
Unknown	38 (7.20%)	690 (5.99%)	
Dual (%)	11 (2.08%)	303 (2.63%)	0.44
Minimally invasive surgery	510 (96.59%)	11,157 (96.91%)	0.68
Charlson comorbidities (%)			
0	345 (65.34%)	7713 (66.99%)	0.58
1	115 (21.78%)	2278 (19.79%)	
2–3	57 (10.80%)	1211 (10.52%)	
> 3	11 (2.08%)	311 (2.70%)	
Year of surgery			< 0.001
2016	25 (4.73%)	1555 (13.51%)	
2017	52 (9.85%)	1689 (14.67%)	
2018	50 (9.47%)	2125 (18.46%)	
2019	68 (12.88%)	2324 (20.19%)	
2020	160 (30.30%)	2613 (18.79%)	
2021 (Q1‐Q3)	173 (32.77%)	1657 (14.39%)	

Abbreviation: SD: Standard deviation.

Among those discharged the same day, the percentage of readmissions was higher (6.8%) compared to those discharged the next day (3.5%) (*p* < 0.01). The most common diagnoses for readmissions were infections (27.0%), gastrointestinal issues (18.95%), and genitourinary‐related complaints (14.2%), such as catheter malfunction (Table 2). However, a composite measure of requiring acute care (i.e., readmission, observation stay, or emergency department visits) was similar between those discharged same‐day vs. next‐day (13.6% vs. 12.3%, respectively; *p* = 0.35). Rates of 30‐day observation stays without readmission were uncommon (NB. % masked due to small cell size; *p* = 0.73), and 30‐day emergency department visits without readmission or observation stay (5.7% vs. 7.8%; *p* = 0.07) were similar in patients discharged the same day and the next day.

**TABLE 2:** Three most common causes of return visits following prostatectomy.

	Readmissions *n* = 438	Observation stays *n* = 119	ED visit *n* = 927
First	Infection	Genitourinary	Genitourinary
*n* (%)	118 (26.94%)	19 (15.97%)	426 (45.95%)
Second	Gastrointestinal	Gastrointestinal	Other
*n* (%)	83 (18.95%)	18 (15.13%)	145 (15.64%)
Third	Genitourinary	Infection	Infection
*n* (%)	62 (14.16%)	14 (11.76%)	102 (11.00%)

*Note:* Most common causes of readmissions, observation stays and ED visits among all patients.

Abbreviation: ED, Emergency department.

After adjusting for patient characteristics, same‐day discharge was significantly associated with increased odds of readmission (OR 1.93; 95% CI: 1.35–2.76; *p* < 0.01) (Table 3). However, there was no difference in the odds of requiring acute care within 30 days of discharge in those discharged the same day compared to those discharged the next day (OR 1.16; 95% CI: 0.90–1.50; *p* = 0.27).

**TABLE 3:** 30‐day outcomes adjusted for year and patient characteristics.

	OR	95% CI	*p*
Discharge			
Next day	Ref		
Same day	1.93	1.35–2.76	< 0.01
Age	1.02	0.99–1.05	0.17
Race			
Black	1.06	0.71–1.57	
Other	0.92	0.52–1.64	
Unknown	0.91	0.60–1.39	
White		Ref	0.66
Dual‐eligible	1.00	0.55–1.81	0.99
Surgical approach			
Open	Ref		
Minimally invasive	1.52	0.78–2.97	0.22
Comorbidities			
0	Ref		
1	1.41	1.12–1.78	0.01
2–3	1.37	1.01–1.84	0.04
> 3	1.55	0.92–2.61	0.10
Year			
2016	Ref		
2017	1.170	0.81–1.70	0.41
2018	0.96	0.66–1.39	0.82
2019	1.07	0.75–1.53	0.70
2020	1.07	0.75–1.53	0.70
2021	1.27	0.89–1.83	0.19

*Note:* Comorbidities defined by Charlson Comorbidity Index.

Abbreviations: CI, Confidence interval; OR, Odds ratio.

## 4 Discussion

This study has three important findings. First, same‐day discharge after prostatectomy for prostate cancer is increasing in the Medicare population. Second, men discharged the same day have nearly double the odds of a readmission within 30 days of discharge compared to those discharged the day after. Third, the need for acute care (i.e., emergency department visits, hospital observation stays and readmissions) following discharge after prostate cancer surgery is relatively common in this population, occurring in more than 10%. However, the odds of requiring acute care within 30 days were similar in the two groups.

With pressures to move patients out of the hospital sooner, not all candidate procedures appear to be amenable to same‐day discharge. For instance, in patients undergoing total knee arthroplasty, those discharged the same day were more likely to experience 90‐day complications requiring readmission (OR: 1.14; *p* < 0.001) [15]. Prior work evaluating same‐day discharge after prostatectomy for prostate cancer has demonstrated its feasibility through case series from high‐volume institutions and surgeons. The benefits of the approach include freeing up hospital beds and lower costs to the healthcare system without compromising safety and patient satisfaction [6, 16, 17]. Generally, in selected patients, same day discharge has not been associated with higher rates of readmission nor reoperation, though the numbers in all studies are small [6, 7, 16]. For example, in the largest single surgeon series of 246 men, emergency room visits and readmissions were rare, both under 2% [16]. Similarly, in a series from two academic medical centers, the odds of complications were similar [6]. In the largest study of data from the National Cancer Database, there was similarly no difference in readmissions or 90‐day outcomes [18]. While these studies clearly demonstrate that appropriate patient selection is possible by some surgeons, they do not inform the generalizability of this ability to others. This is particularly important, given that 60% of surgeons performing the procedure do fewer than five per year, and 30% do only one [19]. One study potentially provides insight into selection. Men undergoing a concurrent lymph node dissection had a 7.8% chance of readmission, compared to just 1.5% in those not undergoing lymphadenectomy, suggesting that these patients may not be well‐suited for same‐day discharge [8].

Our findings should be interpreted in the context of its limitations. First, our study uses national Medicare data, which lacks measures of cancer severity, such as grade and stage. However, we limit the study population to include only those discharged on the same day or next day to exclude those undergoing more extensive procedures due to more advanced disease or due to perioperative complications requiring longer hospitalization. In this homogenous population, we would not expect disease biology to play a significant role in readmission acutely after discharge. Second, the study population is over the age of 65 and our findings are not generalizable to younger patients who may be more amenable to this approach. Nonetheless, prostate cancer occurs commonly in the Medicare population, with an average age at diagnosis of 67 years [10]. Thus, these results are informative for a substantial population burdened by the disease. Finally, associations within the data do not imply causality and the possibility of unmeasured confounding exists. Importantly, one would expect that selection would favor those being discharged the same day. Despite this, our findings suggest a nearly twofold increase in odds of readmission in this group. Further, the analysis controls for patient characteristics known to have relationships with readmission risk, including age, comorbidity and socioeconomic vulnerability, as measured by eligibility for both Medicare and Medicaid. Despite this, some socioeconomic and social vulnerability factors may confound our data.

Nonetheless, this is the first national Medicare study assessing readmission among men being discharged on the same day as their prostatectomy for prostate cancer. Our data suggest that acute care events following next‐day discharges are more likely to involve an emergency department visit without readmission, whereas patients discharged the same day are more likely to experience a return visit that leads to readmission. The higher odds of readmission in the same‐day discharge group are particularly striking given that these patients are likely to be highly selected given the novelty of the same‐day discharge approach. That readmissions following same‐day discharge are higher could reflect a lower threshold to re‐admit a patient who returns to the hospital after same‐day discharge compared to those having an overnight stay. Alternatively, a less robust social support network or other unmeasured social risk factors—factors that may not be assessed by most surgeons—could increase the likelihood of readmission for men discharged on the day of surgery, compared to those who are monitored more closely in the hospital after surgery, as other studies have noted [13]. Finally, poor health literacy and inadequate education may be associated with readmission in those discharged the same‐day, which may be mitigated by attention to process measures [20, 21]. To improve generalizability of same‐day discharge, which is clearly feasible when implemented by certain surgeons, more explicit selection criteria and pathway guidance are warranted. Nonetheless, despite these concerns, rates of same‐day discharge seem to be significantly increasing, likely as a result of efforts to improve bed utilization parameters and new technologies, including single‐port techniques [22].

## 5 Conclusion

In conclusion, patients discharged the same day following prostatectomy had higher odds of readmission compared to those discharged the day after surgery. However, use of acute care within 30 days of discharge was similar between the two cohorts. Further work should continue to seek to better describe factors associated with readmission, and acute care needs more broadly, to aid in patient selection following prostate cancer surgery.

## Author Contributions

Christopher P. Dall, MD: Conceptualization, Formal analysis, Investigation, Methodology, Writing (original draft preparation and review and editing). Xiu Liu, MS: Visualization, Resources, Methodology, Writing (review and editing). Sarah J. Leick, MD: Conceptualization, writing (review and editing). Preeti Chachlani, MS: Conceptualization, methodology. Kassem S. Faraj, MD: Conceptualization, methodology, writing (review and editing). Arnav Srivastava, MD: Conceptualization, methodology, writing (review and editing). Samuel R. Kaufman, MA: Visualization, Resources, Methodology, Writing (review and editing). Vahakn B. Shahinian, MD, MS: Supervision, Writing (review and editing). Brent K. Hollenbeck, MD, MS: Conceptualization, Funding acquisition, Investigation, Methodology, Project administration, Supervision, Validation, Writing (review and editing).

## Funding

American Cancer Society NCI R01 CA269367, NCI R01 CA279746.

## Ethics Statement

We have received institutional review board approval for our data from the Mass General Brigham IRB. Written consent is exempt for this IRB.

## Conflicts of Interest

The authors declare no conflicts of interest.

## Data Availability

Data sharing not applicable to this article as no datasets were generated or analysed during the current study.
